# Between forwarding and mentoring: a qualitative study of recommending medical doctors for international postdoctoral research positions

**DOI:** 10.1186/1472-6920-11-31

**Published:** 2011-06-09

**Authors:** Dario Sambunjak, Matko Marušić

**Affiliations:** 1 1Department for Research in Medicine and Healthcare, University of Split, School of Medicine, Split, Croatia

## Abstract

**Background:**

Young scientists rarely have extensive international connections that could facilitate their mobility. They often rely on their doctoral supervisors and other senior academics, who use their networks to generate opportunities for young scientists to gain international experience and provide the initial trigger for an outward move.

**Methods:**

To explore the process of informal recommending of young physicians from a small country for postdoctoral research positions in foreign countries, we conducted in-depth interviews with eight senior academics who acted as recommenders and eight physicians who, based on the recommendations of senior academics, spent at least a year working in a laboratory abroad. Interviews were transcribed and analyzed by using the framework approach.

**Results:**

The findings showed that recommending can take four distinct forms: 1) forwarding information, 2) passive recommending, 3) active recommending, and 4) mentor recommending. These forms differ in their level of commitment and mutual trust among actors, and possible control over the success of the process. Two groups of recommendees - 'naive' and 'experienced' - can be distinguished based on their previous scientific experience and research collaboration with the recommender. Crucial for the success of the process is an adequate preparation of recommendees' stay abroad, as well as their return and reintegration. The benefits of recommending extend beyond the individual participants to the scientific community and broader society of the sending country.

**Conclusions:**

With a sufficient level of commitment by the actors, informal recommending can be a part of or grow into an all-encompassing developmental relationship equal to mentoring. The importance of senior academics' informal contacts and recommendations in promoting junior scientists' mobility should be acknowledged and encouraged by the research institutions and universities, particularly in developing countries.

## Background

Mobility is one of the key features of scientific careers. The universal nature of science and wide-spread use of English as the language of scientific communication allows people educated in one country to continue their career in another country. In academic communities, international mobility of teachers, researchers and students is considered a prerequisite for continuing participation and access to global science [1]. In many countries, a period of work in another country is necessary for the advancement in a scientific career [2]. The United States is an especially attractive receiving country, not only because of its superior scientific infrastructure and productivity [3], but also due to the regulations that make medical doctors eligible for the postdoctoral positions even without a PhD degree [4].

Young scientists, however, rarely have extensive international connections that could facilitate their mobility. They often rely on their doctoral supervisors and other senior academics, who use their networks to generate opportunities for young scientists to gain international experience and provide the initial trigger for an outward move [5]. Helping to establish connections and networks was found to be one of the important mentoring functions in academic medicine [6].

Melin found that only 15% of young Swedish researchers ('postdocs') who spent some time working abroad had got in contact with their host institution through their supervisor [7]. All participants in that study, however, were recipients of grants from Swedish organizations, which greatly enhanced their chances of independently choosing their 'postdoc' positions. The role of senior academics in connecting and recommending young researchers for a working or training position abroad may be even more important in smaller scientific communities, such as that in Croatia, where the resources for supporting early-career mobility of scientists are meager.

Marušić gave a description and preliminary evaluation of a model of using personal and informal contacts to secure 'postdoc' positions for junior researchers [4]. Based on that model, a number of Croatian physicians during the last two decades was recommended and sent for a period of time to work abroad, mainly in the USA. Due to informal nature of this mobility scheme and sporadic recordkeeping in relation to it, the exact number of junior researchers who used this scheme is difficult to establish. Based on the published data [4] and unpublished communications, we estimate that the total count could be up to several hundred, which is a considerable number relative to the size of Croatian scientific community [8].

The aim of this study was to gain a deeper understanding of the process of recommending young medical doctors for a postdoctoral scientific training in foreign countries.

## Methods

### Participants

The first group of participants were recommenders, defined as senior members of the Croatian academic medical community, who used their contacts and gave their recommendations to help young physicians obtain a scientific training abroad. The second group were recommendees, defined as Croatian physicians who got their scientific training abroad with the help of the recommenders.

We used the snowball method to find the potential participants. To explore the broadest range of experiences possible, we conducted interviews with participants from each of the four medical schools in Croatia and included participants of both genders, from different areas of medicine (basic and clinical sciences), and of varying duration of experience in the role of recommender. We included as recommendees only medical doctors whose training abroad had been planned to be at least a year long.

### Data Collection

Two separate interview guides were developed for recommenders and recommendees, based on three sources: 1) previous literature on mentoring and career mobility, 2) February 2007 online discussion on the investigated topic among Croatian scientists on the Connect Portal at http://www.znanost.org, and 3) pilot interviews with a recommender and a recommendee. Interview guides were designed to uncover two areas of interest: a) modes and dynamics of recommending and b) risks and benefits of recommending. Semi-structured interviews were conducted by the first author between 24 November 2008 and 31 March 2009, face-to-face, in home institutions of the participants, and were audio recorded for further transcription and analysis.

### Analysis and Ethics Approval

Interviews were analyzed using the framework approach, which consists of familiarization with the data, identifying a thematic framework, indexing and charting of the data, and their final mapping and interpretation [9,10]. Visual displays were used to organize and interpret data [11].

The study was approved by the Ethics Committee at the University of Zagreb School of Medicine. All participants were informed about the purpose and the methods of the study and signed their written consent.

## Results

Eight recommenders and eight recommendees were interviewed for this study. All of the recommenders were either associate or full professors; two were female; six worked in preclinical (basic sciences), and two in clinical research and practice. Seven recommendees did their scientific training in the United States and one in Germany; three were females. At the time of study, five were working in preclinical and three in clinical medicine; they went abroad between 1996 and 2004, and stayed there between four months (a participant who cut short his planned 1-year stay) and three years. At the time of leaving for international postdoctoral position, two participants had a substantial scientific experience, while others had either limited or no such experience.

### Modes and Dynamics of Recommending

The findings show that the process of recommending occurs on four levels of intensity, involves two major groups of actors, and evolves in four phases.

#### Levels of recommending

##### Forwarding information

**Figure 1:**
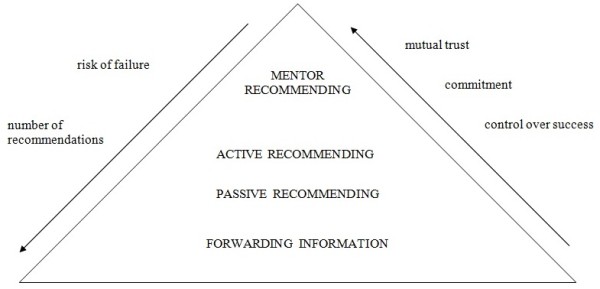
**Levels of recommending**. Mutual trust, commitment and control over success increase from the basic to the highest level, whereas risk of failure and number of possible recommendations increase from the highest to the basic level of recommending.

On the most basic level, senior members of academic community only forward received information about available opportunities, places and scholarships for scientific training abroad. That is usually done by sending an e-mail to the contacts in the address book or posting the information on an institutional web page or forum. If the action remains on this level, no relationship is actually established between the senior scientist and the recommendee, so this can only conditionally be considered recommending. On this level senior scientists can reach a large number of potential recommendees, but they have little or no influence on the response or the quality of candidates and cannot assess the results of their action (Figure 1).

At least once a week I get a mail in which a place [for research work or training] is offered. And then - what do I do? I scan over my address book, forward the mail to 20 or 50 other addresses and the story finishes there. (Recommender 1)

Potential recommendees can use the information and end up in a foreign laboratory even without ever seeing the person who initially forwarded the mail. However, in order to apply for the position abroad, they need to obtain a written recommendation either from the person who forwarded the information or from another senior scientist.

##### Passive recommending

Candidates who want to use the forwarded information or have themselves found an opportunity for scientific training abroad need to approach a senior scientist or a faculty member who is willing to help them by writing a recommendation. This is the first level of recommending in which the two actors have personal contact, which is usually prompted by the potential recommendee.

Many people have asked me to write them a recommendation for some position they found, either themselves or through someone else. (Recommender 2)

The two sides do not have to know each other well, but a basic level of trust is necessary to allow the recommendee to ask for help and the recommender to give it (Figure 1).

[Recommendee] decided for himself and wanted to go to Oxford; I wrote all the necessary recommendations for him. That means, he found the place himself, and I supported him in that... he came to me because he didn't have anyone else to approach. And he knew I had been [trained] abroad, so I would be appreciative. (Recommender 3)

The interaction is in most cases one-off, meaning that after the initial contact and a short assessment of the candidate, which can also be based on the advice from another senior colleague scientist, recommenders write their recommendation, but the relationship does not continue or develop. Senior scientists can also refuse to give the recommendation if they judge the candidate unworthy.

##### Active recommending

On this level, recommenders actively seek appropriate candidates for an available scientific position abroad, and try to encourage and prepare them for such a move.

[Recommender] contacted me and said that he has a place [abroad], gave me some basic information and asked me to think about it and make a decision within the next two weeks. And that, if I'm willing, this sounds to him as a good chance for some additional education and so on... (Recommendee 1)

These candidates usually did not closely collaborate with recommenders, although their acquaintance can be long and established during the candidates' studies. The candidates were mostly recent medical graduates without much experience in either scientific or clinical work ('naive'). Active recommending can be a beginning of mentoring relationship, because it implies a greater level of commitment, responsibility and continuity of care. Mutual expectations are higher than on the previous levels of recommending and this, together with limited time and opportunities for preparation, can increase the risk of disappointment and failure in the process of recommending (Figure 1).

##### Mentor recommending

When there is a previous experience of research collaboration, recommending is a part of a mentoring relationship. The recommendee is 'experienced', usually works in the laboratory or institution of the recommender, their interaction is frequent and purposeful, and their mutual trust significant (Figure 1).

I sent a couple of dozen people for training abroad... They were mostly people who somehow came into research-related contact with me. They were not always members of my immediate research group, but they were all a part of the broader research program through which I came into contact with them. (Recommender 4)

In this type of relationship, preparation for work abroad is longer and more thorough, which reduces the chances of disappointment or failure (Figure 1).

#### Actors

##### Recommender

Personal experience of training abroad, intensive scientific activity, and motivation for recommending are the key features that characterize recommenders. A senior member of academic community must have an international reputation and active network of collaborators to be able to effectively place a younger researcher in a productive scientific group abroad. Sporadic short-term visits to foreign institutions are usually insufficient to build such a network.

At our medical school we don't have many people who have a substantial experience in working abroad. Many people visited foreign institutions, spent a few days there, and that's all... But, that's nothing, you have to work somewhere in order to establish true contacts. (Recommender 3)

Lower levels of recommending ('forwarding information' and 'passive recommending') do not imply a great effort or commitment on the part of recommender. But, higher levels ('active' and 'mentor' recommending) can be time- and energy-consuming, and require some personal involvement. Motivation for recommending can stem from the feeling of altruism, need to build up the capacity of one's own laboratory or the sense of duty.

All these dissertations, these are all my [recommendees]... That makes me happy. (Recommender 5)

There is a selfish component... One actually chooses, tries to get some high-quality people in one's own laboratory. (Recommender 1)

As a project leader, you have the responsibility not only to complete the experiment and publish something, but also to bring up those young people... and make scientists out of them. (Recommender 6)

##### Recommendees

Two groups of recommendees can be distinguished. One group, which we named 'experienced', consisted of physicians who went for a scientific training abroad after they had worked for several years in basic research or clinical medicine. They mostly had a secured job at a school of medicine or in a hospital, and their relationship with their recommender was usually on the level of mentorship. The other group, which we named 'naive', consisted of recent medical graduates or interns. At the time of going abroad they did not have much experience in either basic research or clinical medicine, and were also without a steady job. Their relationship with the recommender began with the act of recommending and was not initially on the level of mentoring.

The most important characteristic of a potential recommendee is an intrinsic motivation for doing science and serious training abroad. Recommenders described this in words such as *enthusiasm*, *interest*, *love for science*, and *ambition*.

[I'm looking for] that spark in the eye which is hard to define, but you can notice it in the people who are interested in science, who want to explore, pose questions. (Recommender 6)

Other desirable characteristics are reliability, diligence, and willingness to sacrifice and accept critique. Recommendee should also have basic communication and social skills. Knowledge of English is advantageous, but not critically important, especially for 'naive' recommendees. The grade point average at the university can be an indication of these characteristics, but not all recommenders considered it as a valid proxy.

If you are motivated and ready for sacrifice, then you'll learn. If you're an excellent student and graduate with the highest marks, and you're not ready for sacrifice, then you have a serious problem. (Recommender 7)

There are some students with perfect grades, yet incapable of working [in laboratory]. They do not have enthusiasm. (Recommender 3)

Whereas the desirable characteristics are basically the same in 'experienced' and 'naive' recommendees, the motivation for going abroad in these two groups can be quite different (Table 1). 'Experienced' recommendees specifically want to expand their scientific knowledge, skills, output and networks. 'Naive' recommendees are often driven by an immediate need to find a job or inspired by a challenge of working in a laboratory or living abroad. A prospect of academic advancement upon return to home country is a significant source of motivation for both groups of recommendees.

**Table 1:** Differences in the process of recommending between the recommendees with the previous scientific or clinical experience and those without it

Aspect of the process	'Experienced' recommendee	'Naive' recommendee
*Phase 1: Establishing contact*		

motivation of recommendee	becoming scientifically independent, building a scientific profile, networking	getting a job, seeing the world, taking a challenge, obtaining material for PhD thesis

*Phase 2: Before going abroad*		

expectance that recommendee should go abroad	high	low

assessment of a potential recommendee	gradual, thorough	quick, superficial

offer to go abroad	expected, planned	unexpected, unplanned

preparation	long	short or none

choice of the place abroad	adjusted to the recommendee	dependant on the offer

*Phase 3: During the stay abroad*		

communication with the recommender	specific, regular	general, sporadic

cooperation with the recommender	continual	rare

duration of stay	shorter (1-2 years)	longer (3 years or more)

productivity of recommendee	High	initially low, gradually increasing

*Phase 4: After return to home country*		

main support by the recommender	obtaining grants and setting up a laboratory	helping to find a job, supervising the PhD thesis

#### Phases

##### Establishing connection

In the first phase, which is short in duration, the two actors come into contact and establish a loose relationship. Recommendees are typically recruited among the final year undergraduates, research fellows, and physicians working in clinical medicine, mostly interns, more rarely residents and specialists. Recommenders continually try to instill their students with enthusiasm for science and, when they know of an open training position abroad, actively search for appropriate candidates.

During my courses, I would always talk how I got my training in the USA, how students can do the same after graduation, and that I would help them. (Recommender 3)

We announce [an open training position abroad] on the faculty council... Spread the word, observe the graduates... I forward the information, for example, through the institutional web pages. (Recommendee 2)

Recommenders use different ways and approaches to reach out and find candidates for scientific career and working abroad.

I put invitations on info boards at the schools of medicine, life sciences and veterinary medicine... Then some people come forward and I interview them... Actually, I'm fishing for good candidates. (Recommender 1)

We announce [that we know of some open positions abroad] at the Faculty Council... Then, by word of mouth... (Recommender 8)

Recommending relationships can also be initiated by the potential recommendee. In some cases, the connecting role is played by an intermediary person, usually a colleague of either recommender or recommendee.

##### Period before going abroad

The second phase is the period before going abroad, which is relatively short (e.g. several weeks) for 'naive' recommendees and much longer for 'experienced' recommendees, who may spend up to several years working in the institution or laboratory of the recommender.

The 'naive' recommenders are usually catapulted to any research position abroad that urgently needs to be filled, after having been assessed by the recommender in one or two interviews, and without much preparation (Table 1). It is mostly a 'take it or leave it' offer by the recommender.

The chance to go abroad came as a surprise... When we knocked at the recommender's door, we were told that there's no job here, but there is - there [in the United States]. (Recommendee 3)

There was not much time for deliberation - 15 days, yes or no. Because they were looking for someone, they needed a person [in the foreign laboratory] and that's it. (Recommendee 4)

The 'experienced' recommendees, on the other hand, were well prepared for their scientific training abroad. Longer research collaboration with recommenders allowed a thorough assessment of recommendees and enough time to select the most appropriate place or laboratory (Table 1).

I have never recommended anyone who did not go through my 'filter', who did not work with me for at least a year... In that way I could get to know their characteristics... (Recommender 4)

When it's about my assistants or collaborators, then I think deeply where I could send them. I talk with them, inquire about their long-term plans... (Recommender 1)

On the levels of active and mentor recommendations, some expectations inevitably occur in this phase, and should be acknowledged and discussed. Recommenders in our study expressed four basic expectations from their recommendees: to accept the offer to go abroad, to eventually return to home country, to achieve some success, and to leave the position available for future candidates. On the other hand, recommendees expected that the recommended position abroad will be appropriate and suitable for their needs and capacities. Furthermore, they expected their recommenders will help them get a job upon the return to home country.

A clear and sincere communication of recommendees' true motives and life plans allows recommenders to take an appropriate action.

I tell them frankly - please, let me know what you want from your lives, so that I know how to handle your case, what to expect from you. Whether you plan to return or stay abroad. And if you will return, would you like to be in basic research or in clinical medicine. I can support any of these plans and desires, but I have to know clearly what I'm supporting. (Recommender 3)

##### Period during the stay abroad

The third phase is the stay in the laboratory abroad, during which the recommendee and recommender communicate with varying intensity, depending on the level of their previous collaboration (Table 1). Although recommenders feel responsibility for the success of the arrangement, their ability to monitor the progress of recommendees is limited. The two actors keep in contact through e-mails, phone calls and personal encounters during recommendees' holiday visits to home country.

A timely announcement of recommendees' plans to return is crucial for their successful reintegration in the home country. The recommender has a responsibility to help with the return.

That was basically a deal - to let them [recommenders] know when we decide to come back. (Recommendee 3)

I tell them - I will do anything I can to help you find the position you want. I think this is a part of the whole story. (Recommender 3)

##### Period after the stay abroad

The fourth phase is the period after recommendees' return from abroad (Table 1). If their work there was productive, they can relatively quickly obtain their doctoral degree or establish their own research group. The support of recommender is especially important for the 'naive' recommendees who do not have any previously established professional connections in the home country.

These young people who returned from the USA... would always want me to supervise their PhD thesis, because of several reasons: first, I knew the subject well enough, because I had worked on similar problems during my stay abroad; second, because they trusted me; and third, because they wanted a supervisor who would understand and accept the values and habits they had developed abroad. (Recommender 7)

The relationship between the actors gradually changes from hierarchical (senior-junior) to collegial (peer). This happens more quickly with the 'experienced' recommendees.

My recommender gave me the freedom here in our department to form my own group and develop independently. Now we function as partners. We consult about some important issues, sometimes even plan some collaborative projects or experiments. (Recommendee 5)

If recommendees decide to stay and settle permanently in the host country, the fourth phase begins with their leaving the laboratory to which they were recommended, usually to go for a clinical specialization or, more rarely, to accept another scientific position abroad.

### Benefits and Risks of Recommending

The primary beneficiaries of the recommending process are recommendees. During their stay abroad, they not only increase their scientific competencies and capacities through work and networking with other scientists, and obtain publications necessary for career advancement, but also go through the process of socialization and acquire the role of a scientist. The experience of living and working in a foreign country can also contribute to the personal growth.

I think that the USA really teaches them life lessons. Not only in the strictly scientific sense... They become independent, they return as completely different persons, persons who know how to lead processes, who are responsible, who have their position in the world. (Recommender 7)

Recommenders also increase their scientific capacity - directly if the recommendees return to their laboratory or institution, or indirectly if they stay in a foreign laboratory and continue their research collaboration with the home country. A history of successful recommendations can enhance the international reputation of recommenders and build their sense of personal accomplishment and contribution to broader society.

My contacts abroad told me literally: we accept anyone you recommend - immediately, without a second thought. (Recommender 1)

[By recommending], you direct the lives of recommendees in an important way and ultimately these people change our country for the better. (Recommender 7)

A steady flux of highly motivated young researchers is a clear benefit for the laboratories abroad. Finally, there are benefits for the sending country's scientific community and the society as a whole. The process of recommending produces young researchers capable of working and competing on the global scientific market. They bring not only international connections and access to new resources, but also a well-developed work ethics and high personal standards of achievement, which can influence their social environment. Even if they stay abroad permanently, they can collaborate with scientists from their home country and ensure the continuation of the recommending process. Therefore, the risk of brain drain is only conditional. The major risk is related to recommendees who fail to demonstrate a basic level of responsibility and social competence during their stay abroad.

One person was really asked to leave, after a month, or month and a half. Because he caused conflicts, offended other people, etc. Such persons can have a toxic effect... For example, to one foreign laboratory we cannot send any more people, we were told: thank you, but not thank you. Don't send us any more people. Because that one person made such a bad mark that they do not want anyone from our part of the world - for a long, long time. (Recommendee 6)

There is also a specific risk of failure for 'naive' recommenders, who are under pressure to learn the science and laboratory work from the very beginning. Coupled with the challenges and allures of living in a foreign country, this pressure makes them a vulnerable group. The failure can manifest as an early and abrupt return to home country or negligible scientific output. Recommenders have a responsibility to realistically present the challenges and opportunities of working abroad, because 'naive' recommendees can easily develop misconceptions and unrealistic expectations.

[Recommendees] were strongly impressed by the recommender, who may have unrealistically depicted the situation [abroad] as overly positive. Much more beneficial than it could ever be. (Recommendee 6)

## Discussion

Senior members of academic community can help young physicians to obtain a working position in a foreign scientific laboratory through several forms of recommending, which differ in their level of commitment and control over the success. Two major groups of recommendees ('naive' and 'experienced') can be distinguished on the basis of their previous working experiences and research collaboration with the recommender. The benefits of the recommending process extend beyond the individual participants to the scientific community and society as a whole. In the context of increased mobility and demand for young scientists [12], but also of ever stronger competition on the global work market, the process of recommending can help in directing young physicians towards those places that will allow a rapid development of their scientific potentials.

By active recommending, senior academics are detecting and mobilizing medical graduates or interns with a serious interest in a scientific career. This function has a great importance, as the majority of countries, particularly those more developed, are experiencing a decline in interest of young people in active involvement with science [13].

Benefits of working abroad accrue to all successful 'postdocs'. Fresh graduates and interns mostly constitute a group of 'naive' recommendees, who begin their education in science by working in a laboratory abroad. During that period, they not only 'learn the ropes' of research, but also produce data and results that allow them to obtain a PhD upon their return to home country. The head of the laboratory abroad, other co-workers, as well as the recommender can all take active part in a 'blended approach' to postgraduate supervision [14]. The process of recommending also contributes to the increase in proportion of successfully completed PhDs, which is relatively low in the countries of scientific periphery [15]. For the 'experienced' recommendees, a period of work in a high-profile foreign laboratory can greatly enhance their career perspectives. A careful selection of the most appropriate place abroad increases the chances of successful transition into autonomous postdoctoral research at a career stage that is particularly prone to attrition [16].

International experience can also bring some less tangible, but no less important benefits in terms of changes in recommendees' personal and professional attitudes [17]. Such changes can indirectly affect the scientific community, medical profession and the broad society in the sending country. Previous studies of highly-skilled workers' migration in transition countries such as Poland have also shown that repatriates contribute not only to the transfer of technology, but also bring with them new techniques of management, as well as a different attitude towards work and working hours, which may be described as a 'capitalist ethos of work' [18]. In post communist societies, which lack a tradition of market economy and democratic institutions, such contributions can have far-reaching consequences. However, the question remains whether the returned recommendees permanently retain the working ethos acquired during their stay abroad.

Our study shows that higher levels of recommending ('active' and 'mentor') imply both finding a research position abroad and taking care that recommendees obtain an adequate job upon their return. This return-facilitating action was not only expected by recommendees, but also felt as a personal responsibility by recommenders. The key role of powerful patrons in securing academic positions for repatriated scientists has been found in some other countries, especially those where scientific opportunities are based more on connections than on transparent and meritocratic processes [2,19]. Recommending may therefore have a negative side-effect of perpetuating elements of corruption in the system of academic placements and promotions.

The risk of 'brain drain' cannot be completely avoided in the process of recommending, especially when the sending countries have relatively good human resources and undergo an economic and developmental progress [20]. However, the alternative to international mobility is not a 'brain gain', but a stagnation or waste of human resources [21]. The most powerful push-factors for migration of Croatian research fellows are the lack of perspective and opportunities for scientific development in the home country [22], and the stay of these young researchers in their home country would in many cases result in their leaving science altogether. Without the active recommending, the group of 'naive' recommendees would probably never get involved in science at all. Recommending and subsequent moves to laboratories in foreign countries can attract and keep young people in scientific careers and bring some benefits to the home country regardless of whether the migrants eventually return or not. It has been observed that highly skilled migrants form 'intellectual diaspora networks' [23] which, if properly managed and harnessed, can be a powerful and useful asset for the sending countries [24].

The limitation of this study was that the sample did not include recommendees who have never returned to their home country. The sample was also limited only to the recommendees who had an experience of either active or mentor recommending. The dynamics of processes on the lower levels of recommending could be explored in future studies.

## Conclusions

The importance of senior academics' informal contacts and recommendations in promoting junior scientists' mobility should be acknowledged and encouraged by research institutions and universities, particularly in developing countries. Efforts should be made to allow an adequate preparation of recommendees for their stay abroad, but also for their timely return, reintegration and continuation of a scientific career in their home country. Very young recommendees without a previous scientific experience are especially vulnerable to the risks of the recommending process and require a special attention and ongoing support. With a sufficient level of commitment by the actors, recommending can be a part of or grow into an all-encompassing developmental relationship equal to mentoring.

## Competing interests

The authors declare that they have no competing interests.

## Authors' contributions

DS and MM designed the study. MM helped in identifying the study participants and organizing interviews. DS conducted and transcribed the interviews. DS and MM analyzed the data and drafted the manuscript. Both authors read and approved the final manuscript.

## Pre-publication history

The pre-publication history for this paper can be accessed here:

http://www.biomedcentral.com/1472-6920/11/31/prepub
